# Department of Practice of Medicine

**Published:** 1893-02

**Authors:** Allen J. Smith

**Affiliations:** Medical Department University of Texas, Galveston


### DEPARTMENT OF PRACTICE OF MEDICINE.

Conducted by Prof. Allen J. Smith, A. M., M. D., Medical Department University of Texas, Galveston.

THE EFFECT OF CHANGE OF POSTURE UPON HEART MURMURS.—Dr. Vincent Y. Bowditch (*International Med. Magazine*, November, 1892) has called attention to the fact that often the intensity of cardiac murmurs, particularly those heard at the base during systole, is accentuated by placing the patient in a recumbent position. From forty-two cases examined with special reference to this point, twenty-one showed an increased intensity when the patient was lying down. Of these twenty-one, nine were basal murmurs, eight were apical, two basal and apical, and three indeterminate. Of the original forty-two cases, five showed increased intensity of murmur when the patient was sitting up; two of these five were apical, three indeterminate and diffuse. Of the original number, the remaining sixteen cases failed to show any special difference in the murmurs on change of position; of these six were basal, nine were apical, and one indeterminate. The author refers to a paper published by Dr. O. B. Campbell (*Medical Record*, June 11, 1892), in which the latter states that of one hundred cases studied in this particular, seventy-eight showed increase of intensity of the murmurs when in the recumbent position, six showed a similar increase when in the upright position, and twelve were not affected by change of position. Bowditch is disposed to regard variation in the position of the heart in relation to the direction of the aorta and to the chest walls, as a factor in causing this difference in the intensity of these adventitious sounds in different body-postures, but acknowledges the insufficiency of this explanation for all cases.

---

THE ALBUMINURIA AND BRIGHT'S DISEASE OF URIC ACID AND OF OXALURIA.—Dr. J. M. DaCosta (*American Journal of Medical Sciences*, January, 1893), in a recent address before the Pathological Society of Philadelphia, after detailing a number of examples of albuminuria occurring in patients whose urine was found to contain excesses of uric acid or of calcium oxalate, urged the curability of the albuminuria of these conditions. He believes that if it be permitted to continue indefinitely, however, it will eventuate in actual disease of the kidney of the form of chronic intestinal nephritis. Many of the so-called "functional" albuminurial are, in his opinion, to be properly classed with that of uric acid and oxaluria, now grouped, perhaps, as "albuminurias of adolescence," as "dietetic albuminuria," or, "postural albuminuria." The underlying condition of renal irritation and congestion induced by these crystalline substances in their excretion is, for a time, at least, amenable to treatment, but eventually passes into actual formative inflammation. As signs of importance in the recognition of the albuminuria of this form from actual Bright's disease, Dr. DaCosta insists upon frequency of dyspeptic phenomena, the absence of hypertrophy, and other cardiac symptoms common in the latter affection, the absence of dropsy and of retinal symptoms, a slight rise of temperature in the afternoons, prominence of such nervous symptoms as listlessness, fatigue, forgetfulness, sleeplessness, headache, and giddiness, and the existence of certain differences in the urinary symptoms from those of Bright's disease. These differences are to be seen in the high specific gravity as contrasted with the persistently low specific gravity in contracted kidney; in the increase of the total solids—especially contrasting in case of the chlorides with a diminution in Bright's disease; in the slight and varying excretion of albumin, and in the infrequence and persistent hyaline type of casts. The prognosis of the condition is, according to the writer, favorable; although the individual case may last for several years, with occasional reappearance of albumin.

The treatment laid down by the author is, in the main, parallel with that of contracted kidney. The extra labor thrown upon the kidneys is to be lightened by calling the various emunctories into play, and by strict care in diet. In the matter of diet, the greatest care is to be exercised. Vegetables, generally speaking, are to be freely permitted; meats containing much nitrogen are to be almost entirely avoided; milk may be used, but is of less consequence than in actual renal cirrhosis. The free use of pure water and of the mildly diuretic waters, hot at bed time and in the mornings should be strongly advised. Alcoholics are to be interdicted. The use of baths, not too cold, with friction of the skin, and free open-air exercise, are clearly indicated. Among drugs, laxatives are indicated, phosphate of sodium, cream of tartar, Rochelle salts. A course of muriate of ammonium, or of iron, is recommended, from time to time. Where the oxalates are present, nitro-muriatic acid is the most promising remedy, and the acids are not unavailable in clearing the blood of urid acid, as shown by Haig.

---

DYSENTERY AS IT OCCURS IN NICARAGUA *Therapeutic Gazette*, November, 1892)—Dr. Judson Daland describes three varieties of dysentery, as met with in the above locality, a *malarial* form, the common *endemic* dysentery, and *epidemic* dysentery. In the first of these forms, spoken of as malarial by the citizens of the locality, there occur pains in the back, head, and about the umbilicus, associated with diarrhœa. There is moderate elevation of temperature. The stools are at first almost entirely composed of mucus, but soon become streaked with blood; they are attended with tenesmus. Hepatic engorgement and actual inflammation, with jaundice, not infrequently occur; but hepatic abscess is a rare complication of this class of cases. Many of these cases are followed by marked anæmia and debility, often lasting for months. Treated early, as a rule, a favorable outcome is reached. Quinine is given (gr. vj), morning and evening; and the following dose is repeated every two hours:

R Ammonium chloride . . . . . . . . . . gr. v
Powdered ipecacuanha . . . . . . . . . gr. v
Tincture of opium . . . . . . . . . gtt. x–xv

Where there is much pain, the laudanum is increased, or morphine is added; and in cases of debility, carbonate of ammonium is substituted for the chloride. The diet is kept light, and water (not iced-water) is freely permitted.

The second form is quite like the above, but much milder, and the pains and fever are practically absent. The stools are composed of fœces mixed with mucus and blood, and the griping is less marked than in the malarial form. The treatment is the same as in the former variety, save that the quinine is omitted.

The epidemic form comes on suddenly, is attended with violent pains in the back, head, extremities and abdomen. The temperature is high from early in the case. The stools quickly become bloody, and are apt to be almost entirely composed of blood. There may be as many as 150 evacuations in twenty-four hours, and in average cases there is generally one for each hour. Severe cases die within a week; favorable cases may continue for several weeks. In some cases, especially children, nervous symptoms supervene, delirium, coma, twitching of the muscles, rolling of the eyes and a tendency to bury the head in the pillow. Hepatic inflammation and suppuration are frequent complications, and are usually fatal. Croupous pneumonia may occur as a complication, also generally fatal. Severe and fatal hemorrhages may terminate the course. The treatment usually followed includes the above remedies, the quinine being increased two or three times. Where these remedies are not retained, because of the gastric irritability, the quinine is continued, but the ammonium and ipecac mixture is replaced by bismuth or tannic acid (gr. xv). If these astringents are of no avail, acetate of lead (gr. ij–iij) or nitrate of silver (gr. $\frac{1}{8}$–$\frac{1}{6}$) is employed. Stimulants are generally required, the best results being usually obtained from sherry, port, or any of the red or white wines, associated with carbonate of ammonium. Food is restricted to milk, lime-water, sago and farina. With careful treatment, the dangerous complications may be usually avoided, and the mortality kept low.

Most cases of malarial dysentery are met in Nicaragua in December, January and February, while epidemic cases are most frequent in March, April and May. Nevertheless, individual cases may be met at all times. The contagiousnes of the epidemic variety, is recognized; and isolation, the free use of carbolic acid, burial of all discharges, and burning or disinfection of soiled linen are general measures.

Dr. Daland, who acknowledges his indebtedness to Dr. Bermudez, of Nicaragua, for the matter presented, states that no work has been done in these cases looking to the discovery of the actual cause, as the *amœba coli;* and the differentiation into the above classes rests upon clinical observation alone. He calls especial attention on the part of physicians of the Southern United States, to which the disease might most easily travel, to the confidence placed by Ncaraguan physicians in the use of chloride of ammonium.

